# How to Improve SBRT Outcomes in NSCLC: From Pre-Clinical Modeling to Successful Clinical Translation

**DOI:** 10.3390/cancers14071705

**Published:** 2022-03-27

**Authors:** Marina Milic, Michele Mondini, Eric Deutsch

**Affiliations:** 1 Gustave Roussy, Université Paris-Saclay, INSERM U1030, F-94805 Villejuif, France; marina.milic@gustaveroussy.fr; 2 Gustave Roussy, Département d’Oncologie-Radiothérapie, F-94805 Villejuif, France

**Keywords:** non-small-cell lung cancer, radiotherapy, SBRT, pre-clinical models

## Abstract

**Simple Summary:**

Despite major research and clinical efforts, lung cancer remains the leading cause of cancer-related death. Stereotactic body radiotherapy (SBRT) has emerged as a major treatment modality for lung cancer in the last decade. Additional research is needed to elucidate underlying mechanisms of resistance and to develop improved therapeutic strategies. Clinical progress relies on accurate preclinical modelling of human disease in order to yield clinically meaningful results; however, successful translation of pre-clinical research is still lagging behind. In this review, we summarize the major clinical developments of radiation therapy for non-small-cell lung cancer (NSCLC), and we discuss the pre-clinical research models at our disposal, highlighting ongoing translational challenges and future perspectives.

**Abstract:**

Despite major research and clinical efforts, lung cancer remains the leading cause of cancer-related death. While the delivery of conformal radiotherapy and image guidance of stereotactic body radiotherapy (SBRT) have revolutionized the treatment of early-stage non-small-cell lung cancer (NSCLC), additional research is needed to elucidate underlying mechanisms of resistance and identify novel therapeutic combinations. Clinical progress relies on the successful translation of pre-clinical work, which so far has not always yielded expected results. Improved clinical modelling involves characterizing the preclinical models and selecting appropriate experimental designs that faithfully mimic precise clinical scenarios. Here, we review the current role of SBRT and the scope of pre-clinical armamentarium at our disposal to improve successful clinical translation of pre-clinical research in the radiation oncology of NSCLC.

## 1. Introduction

Despite major research and clinical efforts, lung cancer remains the leading cause of cancer-related death. While the delivery of conformal radiotherapy and image guidance of stereotactic body radiotherapy (SBRT) have revolutionized the treatment of early-stage non-small-cell lung cancer (NSCLC), additional research is needed to elucidate underlying mechanisms of resistance and identify novel therapeutic combinations. Clinical progress relies on the successful translation of pre-clinical work, which so far has not always yielded expected results. Improved clinical modelling involves understanding our models and selecting appropriate experimental designs that faithfully mimic precise clinical scenarios. Here, we review the current role of SBRT and the scope of pre-clinical armamentarium at our disposal to improve the successful clinical translation of pre-clinical research in the radiation oncology of NSCLC.

## 2. Place of SBRT in the Treatment of NSCLC

The fundamental difference between SBRT and conventional radiotherapy is that SBRT allows the delivery of ablative doses in 1 to 5 fractions with high conformal techniques. A typical SBRT course of stage I disease delivers 54 Gy in three fractions over 1 week.

### 2.1. Early-Stage NSCLC

SBRT has established itself as the standard of care in peripheral stage I disease in those patients who are not medically fit for surgery. The high tolerability rate and the outpatient nature of treatment make it a highly appealing treatment option without compromising local tumor control rates, which exceed 90%.

Whether SBRT should be offered as an alternative to surgery to those patients who are medically fit remains a matter of debate [1] as most data originate from retrospective or non-randomized studies. Findings from the single-arm phase 2 NRG Oncology RTOG0618 Trial [2] involving operable early-stage patients suggest a favorable 96% local control rate and treatment-related morbidity, supporting the need for further phase 3 randomized trials. That said, carrying out phase 3 trials has proven challenging. A pooled analysis by Chang et al. [3] of two phase 3 STARS (NCT00840749) and ROSEL (NCT00687986) trials that were closed prematurely due to poor accrual showed a 3-year overall survival of 95% vs. 79% favoring SBRT. Additionally, a better quality of life was reported [4] in the SBRT arm.

However, high-quality level 1 evidence is still scarce and recent randomized trials comparing SBRT vs. surgery including SABRTooth (NCT02629458) have failed to bring such evidence, partly due to barriers to recruitment and intrinsic patient preferences. Nonetheless, several phase 3 or randomized trials are on the way, including JoLT-Ca/STABLE-MATES (NCT02468024), VALOR (NCT02984761) and POSTILV (NCT03833154).

### 2.2. Oligometastatic Disease

Patients with oligometastatic disease [5,6], meaning a limited number of metastases in a limited number of organs (typically less than five in 1 to 3 organs), represent an increasingly important subset that can benefit from the addition of SBRT to systemic treatment [7]. They represent a subset of patients in which we can aim to achieve long-term survival or even cure. SBRT has been increasingly integrated into the treatment schemes of selected oligometastatic patients as an alternative to surgery. Data from multiple early-phase studies have shown that SBRT is a technically feasible, low-toxicity and highly effective local therapy (70–90% local control) for patients with metastasis in the lung, liver, spine, brain or multiple sites. Local control using SBRT was achieved across tumor types including colorectal [8], breast [9], NSCLC [10] and other rather radioresistant types such as sarcoma, renal cell and melanoma [11].

In NSCLC, most trials investigating systemic treatments do not stratify patients by the number of lesions, leading to a wide range of PFS and OS [12]. It is only recently that trials have started looking into the benefits of SBRT in NSCLC patients. In a prospective phase 2 study, De Ruysscher et al. [13] enrolled metastatic NSCLC patients with <5 synchronous lesions treated with SBRT showing a median overall survival (OS) of 13.5 months and a median PFS of 12.1 months. Only two patients (5%) had local recurrence. The treatment was well tolerated, highlighting a favorable subgroup of NSCLC with synchronous oligometastasis who might benefit from radical treatment. Similarly, Salama et al. [14] included patients with five or less NSCLC lesions administering a dose of 50 Gy in five fractions. Median OS and PFS were 22.7 and 7.6 months. A worse PFS was observed in patients who had more than two sites treated with SBRT, adenocarcinoma histology or progression after systemic therapy. These data are to be taken with caution due to the single-arm nature of studies and the scarcity of randomized level 1 evidence data. Palma et al. rightly argue that non-randomized data suggest that ablative treatment is feasible and able to achieve local control in patients with liver, lung, spine or brain metastasis and even at multiple organ sites. When used in patients with CRC hepatic metastasectomy, SABR led to a nearly 50% 5-year survival but was as low as 15% in the less favorable risk factors patient group [15]. Ablative treatment of adrenal metastasis led to a 25% 5-year survival in NSCLC patients [16], while treatment of hepatic metastasis in breast cancer patients resulted in a 22% 5-year survival [17].

Additionally, some of the studies included multiple tumor histologies with different curability rates and behaviors. Even though the survival data reported are encouraging and sometimes better than anticipated, one should also ask the question of selection bias based on favorable inclusion criteria [18] and support urgent prospective randomized trials.

## 3. From Bench to Bedside: Pre-Clinical Models and Their Challenges

As discussed above, the field of radiation oncology has rapidly evolved in terms of both the understanding of radiobiology and technical advances including image-guided, intensity-modulated and stereotactic radiotherapy. We should rightly ask ourselves the question: have all those advances translated into clinical benefit? If not, why? Indeed, the harvesting and successful translation of discoveries relies on the development of advanced preclinical models that reflect clinical scenarios both in terms of radiation exposure conditions and biological responses [19,20].

While SBRT has begun to revolutionize the clinical management of patients, much remains to be investigated in the preclinical setting in order to reveal its full potential. Recent evidence highlights a different underlying radiobiology of high dose per fraction to that of conventionally fractionated radiotherapy [21,22,23]. Therefore, there is an urgent need for appropriate preclinical models that are able to accurately mimic the clinical use of SBRT and provide reliable and translational information on radiobiology, efficacy, combinations and toxicities.

The current poor performance of many investigational treatments suggests that the preclinical models used so far to investigate the efficacy of SBRT lack clinical predictive power. One of the explanations is the lack of preclinical models that truly recapitulate human disease heterogeneity and complexity. The scientific community has attempted to address this issue with the development of increasingly complex models [24], some of which are reviewed below. It has become clear over the last decades that a significant mismatch exists between data generated in preclinical in vitro and in vivo models and successful clinical translation (Johnson et al.). The failure of preclinical models to recapitulate patients’ tumor heterogeneity and complexity is the most cited reason.

## 4. In Vitro Models

### 4.1. Cell Lines

Even though cell culture has immensely contributed to expanding our knowledge of cancer biology, the translation of in vitro data into clinical practice has been inconsistent. Many different reasons have been put forward to account for this inconsistency. First, cell lines are difficult to derive from patients’ tumors and, when expanded, develop outside their natural tumor microenvironment in an artificial normoxic environment that is rich in glucose and growth-factors, which selects for a nearly clonal subpopulation of rapidly-growing cells, neutralizing the initial cell heterogeneity [25]. In a primary xenograft model of small-cell lung cancer (SCLC), Daniel et al. [26] compared gene expression within the xenograft model, identifying a group of tumor-specific genes expressed in primary SCLC and xenografts that was lost during the transition to tissue culture and that was not regained when the tumors were re-established as secondary xenografts. It can be reasonably argued that such genetic divergence may be a common feature of many cancer cell culture systems and their primary tumors, highlighting the functional limitations of such models in preclinical development. As discussed above, solid evidence indicates that the genetic divergence between a primary tumor and the derived cell line is greater than that of a direct xenograft [27].

Determining cancer cell radiosensitivity using clonogenic assays has been the gold standard approach over last few decades, and it was shown to be relevant to the tumor response to irradiation [28]. One of the first links between oncogenes and radioresistance was established with the KRAS oncogene more than two decades ago. RAS activation was shown to increase clonogenic survival and decrease tumor growth delay following irradiation [29,30,31]. However, it is only recently that the association of genetic profiles such as EGFR mutational status in NSCLC on clonogenic survival parameters has been described [32,33]. Similarly, cell lines have been used to investigate the role of p53 in sensitivity to radiation. It has been generally admitted that p53 is required for radiation-induced apoptosis. Hu et al. [34] showed that H460 wild-type cells were more radiosensitive than their p53 null (H460crp53) counterparts but that this differential response was due to increased senescence rather than apoptosis. Considering that radiosensitivity is influenced by other concomitant genetic alterations, preclinical models integrating the inherent genetic profile complexity are needed.

### 4.2. Ex Vivo Tumor Models

First described in 1993 by Benali et al. [35], lung organoids have been subsequently established to model cystic fibrosis or bronchiolitis and, more recently, COVID-19 (SARS-CoV-2) infection. Different types of organoids exist, including tissue-derived, embryonic stem-cell-derived and induced pluripotent stem-cell-derived organoids. The successful three-dimensional culture of patient-derived NSCLC organoids was reported in 2013 by Endo et al. [36], with an 80% success rate being achieved using matrigel. However, it was not before 2019 that a protocol for long-term expansion was described by Sachs et al. [37] One of the challenges inherent to the long-term expansion of NSCLC organoids is the overgrowth by normal airway tissue over primary NSCLC as tumor cells lack a selective advantage in the organoid model, leading potentially to the loss of certain NSCLC subtypes. This has also been reported in colorectal and prostate cancers [38]. Organoids have been proposed to be a better in vitro model than 2D cell lines due to higher rates of preservation of histologic and molecular characteristics of their parental tumors.

Patient-derived organoids have contributed to successful drug screenings, showing concordance with matched patient tumors [39,40]. Until now, only a few reports of primary NSCLC organoids for drug screening have been published. Sachs et al. showed differential responses of NSCLC organoids to conventional chemotherapy agents including cisplatin and paclitaxel and reproduced tyrosine kinase inhibitors (TKI) sensitivity in ERBB2-mutant organoids. Recently, Li et al. [41] demonstrated the feasibility of a high-throughput drug response screening using 24 drugs with a consistent drug-response profile to parental NSCLC.

Organoid technology offers new opportunities to study tumor immunobiology and is rapidly adapting to cancer modelling [42]. Organoid cultures using native or reconstituted tumor micro environment (TME) components have the potential to provide valuable information on the role of the TME immune components in cancer development and progression. Tumor organoids recapitulating the immune TME or immune-organoids offer promising future applications including the testing of immunotherapy agents as well as personalized cancer immunotherapy [43].

Despite those novel approaches, a direct clinical application for personalized treatment is still awaited. Some of the challenges that will have to be faced include the availability of sufficient tissue in patients’ samples and establishing sufficiently fast-growing organoids in order to inform treatment decisions on time.

### 4.3. In Vivo Models

There is no doubt that animal models represent invaluable experimental tools and have played a key role over decades in the advances made in radiation biology. One of the first example is the use of the ram testicular model by Regaud and Nogier in 1911 [44] to investigate the ability of fractionation to spare normal tissue. In the oncology discovery race, the mouse model has established itself as the gold standard tool to study tumor response and novel drug–radiation combinations.

#### 4.3.1. Traditional Xenograft Models

Xenotransplantation is the process of transplanting living cells or tissues to another species. Traditional xenograft mouse models involving transplantation of human immortalized cell lines engrafted into immunocompromised mice have been the cornerstone of research. Human cancer cells can be injected subcutaneously, orthotopically or systemically by intravenous injection. They involve the use of athymic nude mice or severe-compromised immunodeficient (SCID) mice. Several cell lines are currently used to model the lung adenocarcinoma response to treatment, including A549, H1975, HCC406 and HCC827, with A549 carrying the highest engraftment rate. The NCI-H226 line is commonly used to model squamous-cell carcinoma and carries a variable engraftment rate. The vast majority of those models use subcutaneous implantation sites (Table 1), representing a major drawback, as discussed later in this paper.

Traditional xenograft models harbor several limitations, including a significant loss of heterogeneity of human cancers. As tumor cell lines are often grown in vitro for many years, they are likely to develop genotypic and phenotypic alterations [60] and, more importantly, such models fail to reproduce human TME. It is now widely accepted that, at least in part, the efficacy of radiotherapy relies on the host immune response. Four decades ago, Stone et al. [61] demonstrated that the tumor response to irradiation was impaired in the absence of a normal T-cell repertoire. They used a syngeneic mouse model, showing a drastic difference in radio-sensitivity where T-cell-deficient mice required over 60 Gy to achieve a comparable tumor control to immunocompetent mice who received 30 Gy.

Since then, extensive evidence has accumulated regarding the involvement of the immune system in the response to RT [62]. These observations highlight the importance of the use of immunocompetent animal models to study the antitumoral effect of radiotherapy.

The widespread use of immune-deficient animals, together with the other factors enlisted above, have resulted in the low success of clinical translation, approaching a failure rate of 85% in early-stage clinical trials [63]. Wong et al. [64] estimated the probability of success of each clinical phase across multiple indications, concluding that 13.8% of all drug development programs lead to approval. However, this figure was only 3.4% for oncology drugs, which was even lower than previously estimated [65].

#### 4.3.2. Patient-Derived Xenograft Models

In an attempt to improve preclinical models and clinical translation rates, patient-derived xenograft models (PDX) have been developed. PDX models allow the transplantation of fresh patient tumor samples or cell suspensions but this comes with a cost: needing increasingly immuno-deficient hosts to prevent rejection. Many different mouse strains were developed over time in an attempt to increase the low take rate associated with human tissue transplantation as opposed to immortalized cell lines. They have widened the horizons of possible preclinical models and rendered previously difficult engraftments possible. Early generations of genetically determined immuno-deficient mice harbor a single mutation and confer modest immune dysfunction. They include nude [66], severe combined immunodeficient (SCID) [67], non-obese diabetic/SCID (NOD/SCID) [68] or RAG-1 null [69] or RAG-2 null [70] mice. A step further was the development of mice carrying a deletion or truncation of the common gamma chain/II2rg [71], who completely lack NK activity, for example, with new complex immunodeficient models emerging constantly. However, careful attention should be paid to the choice of experimental irradiation models: when it comes to their relevance in radiation oncology, one should bear in mind that some immunodeficient models are inherently radiosensitive due to impaired double strand breaks (DSB) repair capacities. Rübe et al. [72] showed distinct yH2AX-foci kinetics in various immunodeficient mouse strains characterized by different genetically defined DSB repair capacities. In addition, using SCID mice that lack functional lymphocytes and have heightened sensitivity to ionizing irradiation [73] with a LD50/30 of 3 Gy might not be the preferred experimental tool in view of the key role of lymphocytes in the immune response to radiation. Importantly, the vast majority of xenograft models use the subcutaneous site of injection and studies involving orthotopic implantation are rare.

One of the advantages of PDX models is that they retain the characteristics of the primary patient tumors, including the histological characteristics and architectures, gene expression profiles, and molecular and tumor heterogeneity [74], and are to date one of the most reliable in vivo human cancer models displaying the most concordant drug response profile to human cancer. A good illustration is a recent study by Crystal et al. [75] demonstrating the utility of establishing in vivo PDX NSCLC models directly from patients’ biopsy specimens for identifying new drug combinations in a model of acquired drug resistance. On the other hand, in PDX models, the tumor stroma, including the vasculature, is that of the host (mouse), and therefore does not reflect the human tumor situation. This makes it difficult to faithfully evaluate tumor and stroma interactions that are key to response radiation therapy but also to combinations with drugs. Additionally, a “murine drift” was described in severely immunocompromised mice, where human tumors became more mouse-like with repeat passaging [27]. 

#### 4.3.3. Syngeneic Immunocompetent Mouse Models

Syngeneic models involve the injection of murine tumor cell lines that are grown and expanded in vitro into immunocompetent animals. This presents the major advantage of preserving an intact murine immune system, making them the only available choice to test immunomodulatory drugs in vivo. Nevertheless, they also present major drawbacks, such as the lack of mutational and microenvironmental heterogeneity, as seen in human cancers: the cell lines used a lack of mutational patterns that recapitulated human intra-patient genomic heterogeneity and were implanted into a limited number of inbred mouse strains that lacked inter-patient heterogeneity. Additionally, the vast majority of syngeneic models are injected subcutaneously, failing to reproduce the complex architecture associated with de novo tumor growth and the natural development of the tumor microenvironment.

Only two C57BL/6-derived murine lung cancer cell lines are currently commercially available. One of them is the Lewis Lung Carcinoma (LLC) established in 1951 from the lung of a C57BL/6 mouse bearing a tumor established from the implantation of primary Lewis Lung Carcinoma [76]. The second is the CMT64 cell line (and its derivative CMT167 line sub cloned for metastatic potential) derived from a spontaneous lung tumor [77]. Recently, some GEMM-derived cell lines have emerged, such as KRASG12D p53−/−, forming lung tumors after intravenous injection, but also giving rise to a metastatic model. In 2020, Nolan et al. [78] were able to develop six new lines capable of forming orthotopic tumors in 75% of recipient C57BL/6 host lungs. While those lines will need further validation, such initiatives are very much needed. 

#### 4.3.4. Orthotopic Mouse Models

While subcutaneous injection is easy to perform, it does not allow the simulation of the natural history of cancer dissemination as no lymphatic or hematogenous metastatic extension is occurring. More importantly, it is associated with a low translation rate, as evidenced by the high rate of negative clinical trials [79]. 

Orthotopic implantation involves the engraftment of tumor cells into the relevant organ of tumor origin or metastatic site, preserving microenvironmental interactions and offering appropriate microvasculature and angiogenesis for tumor growth. The tumor microenvironment is crucial in tumor development and response to treatment [80] and recent evidence suggests that it varies with the anatomical site of implantation. Devaud et al. [81] observed that despite injecting matched cancer cells, the same tumors implanted in different anatomical sites varied in their response to immunotherapy and differed in their microenvironment. In addition, the surrounding normal tissue at the site of implantation plays a key role in shaping the TME composition, and by extension, the response to treatment. Growing evidence suggests that the TME itself is involved in the initiation and progression of primary lung carcinoma [82,83]. Another important characteristic not to be overlooked is hypoxia, a key feature of solid tumors significantly affecting the sensitivity to radiation therapy and, as a result, clinical outcomes including tumor progression, likelihood to metastasize and overall survival [84,85]. Preclinical modelling ought to take into account that different animal models can yield different hypoxic profiles [86] and influence treatment outcomes. Graves et al. studied hypoxia in A549 human lung adenocarcinoma orthotopic and subcutaneous models, showing the presence of hypoxia in the heterotopic model, while hypoxia was minimal in orthotopic models [87].

All these data suggest that the site of implantation matters and careful attention should be paid when considering which model is best suited to replicate individual human scenarios and assess responses to individual treatment modalities. Another challenge in orthotopic lung models is the development of solitary nodules with no regional or metastatic dissemination simulating the clinical features of human lung cancer and clinically relevant treatment modalities including SBRT. 

The traditional implantation route for orthotopic models is surgical transpleural or percutaneous, allowing the induction of localized tumors with multiple nodules such as the model developed by our group [58,88]. Recently, Nakajima et al. [89] established an orthotopic lung cancer model by means of a non-surgical transbronchial approach in nude mice using human NSCLC lines, establishing a clinically relevant model human xenograft model bearing a solitary nodule.

To allow the generation of more reliable preclinical data, the use of such orthotopic models should be expanded in the forthcoming years.

#### 4.3.5. Genetically Engineered Mouse Models (GEMMs)

GEMM models have been developed using gene targeting by inserting targeted donor constructs into embryonic stem cells of mice. These embryonic stem cells, containing the desired gene mutation, are then injected into recipient mice blastocysts and implanted into pregnant females [90]. This complex process was recently facilitated by the development of genome-editing tools such as the CRISPR/Cas9 systems, allowing the insertion of targeted mutations into the mouse germ line [91] and the study of gene function in vivo. 

GEMMs models develop de novo spontaneous tumors due to the oncogene activation or somatic inactivation of tumor suppressor genes in a natural immune-proficient microenvironment. Tumors developing in GEMMs accurately mimic histological and molecular features of human cancers but also reasonably preserve genetic heterogeneity. In this way, they are superior models to cancer cell inoculation models, which can also be metastatic in nature from the start. They are a useful model to study tumor responses to radiotherapy in specifically defined genomic backgrounds that are seen in human tumors. Recently, successful models of AIJ-SPC-TP53-273H transgenic mice allowed the exploration of the oncogenic potential of TP53 gene in the spontaneous development of lung adenocarcinoma [92]. Importantly, cancer development in these mice had a latency period and was associated with other genetic alterations that are similar to human adenocarcinoma. Equally, they allow the study of radiation-induced carcinogenesis and normal tissues. GEMMs have been successfully used in assessing radio-sensitizers and, more recently, immune checkpoint inhibitors in a KRAS-mutant NSCLC model [93]. 

However, as with all models, GEM models have limitations. Several mutations are often introduced simultaneously, not mimicking the sequential acquisition of mutations in the multistep oncogenesis of human cancers, which can have a significant impact on tumor evolution and modelling. For example, in a GEM model with simultaneous activation of K-Ras and inactivation of p53, the tumor would undergo lower evolutionary pressure and present less genetic complexity than if sequential events were happening. While this can be overcome by using different recombination systems, we should bear in mind that GEM-derived tumors are not human tumors. They are developed within months, whereas human tumors may take years or sometimes decades and carry less genetic aberrations and complexity. Another challenge lies in the ability of GEMs to model metastasis [94]. However, more importantly, GEMs often develop multiple spontaneous tumors at different sites, limiting the applicability to SBRT where a precise delineated radiation is applied to the target lesion. Another drawback is that developing GEMMs models requires significant time, money and expertise.

Nonetheless, GEMMs represent valuable tools in cancer research. They have the advantage of offering preserved microenvironmental, genetic and histological features and, therefore, can be predictive of human tumor response. In pre-clinical radiation research, they are an interesting tool, allowing the preservation of an immunocompetent host and the study of tumor–stroma interactions. The main pros and cons of preclinical models discussed are summarized in Table 2 below.

## 5. Discussion and Future Perspectives

### 5.1. Drug Combinations with SBRT

The immunomodulatory effects of SBRT have revealed a promising potential world of synergy with immune-modulatory agents, starting with immune checkpoint inhibitors in the treatment of NSCLC.

#### 5.1.1. Immune-Checkpoint Inhibitors 

Mounting evidence suggests that radiotherapy combined with PD-1/PD-L1 inhibitors can improve immunosuppression and restore CTL responses, leading to tumor growth suppression and improved survival. Radio-immunotherapy combinations are offering new perspectives from early stage to oligo and metastatic disease spectrums.

Current clinical practice in advanced NSCLC is largely based on results from the phase 3 PACIFIC trial [95], which demonstrated the benefit of consolidation therapy with the PD-L1 inhibitor Durvalumab in patients who did not have disease progression after two or more cycles of chemo-radiotherapy. Patients treated in the Durvalumab arm had a median PFS (Progression-Free Survival) of 16.8 months vs. 5.6 months and an overall response rate (ORR) of 28.4% vs. 16.0% in the placebo arm. Similarly, an ongoing phase 2 Hooiser Cancer Research Study, LUN14-179 [96](NCT02343952), looking at the benefit of consolidation pembrolizumab following concurrent chemo-radiotherapy in patients with unresectable stage III NSCLC has shown promising results, with a mPFS of 18.7 months. It is becoming increasingly clear that SBRT has the features of a key partner in adjuvant immunotherapy. SBRT has the advantage of sparing radiation-induced lymphopenia as compared to conventionally fractionated radiation, a key consideration considering the increasing importance of the immune system in antitumoral response and in combination with immune-checkpoints [97,98]. Currently, more than 100 trials combining SBRT and anti-PD-1/PD-L1 are ongoing despite limited knowledge on how dose and fractionation schedules or timings affect antitumor responses. The majority of available data are currently from retrospective or small-size cohorts, meaning that the optimal sequencing and time window for combination treatment are still to be determined, as discussed later. 

#### 5.1.2. SMAC Mimetics

SMAC (second mitochondria-derived activator of caspase) is a pro-apoptotic mitochondrial protein that is an endogenous inhibitor of a family of cellular proteins called the Inhibitor of Apoptosis Proteins (IAPs). SMAC mimetics (SMs) are promising new agents that are progressing from bench to bedside. They induce cancer cell death predominantly via a cIAP-dependent mechanism regulated by death receptor ligands [99] acting as sensitizers and reducing the threshold for cell death induced by chemo or radiotherapy. Eight new molecules have been tested in clinical trials and proven to be well tolerated and, importantly, non-toxic towards healthy tissue. However, their clinical efficacy as monotherapies is limited. Recent data indicate that tumors must be able to produce and respond to tumor necrosis factor alpha (TNFα) in order for SMs to exert their anti-tumor effect. Tumors that do not fulfil the previous TNFα criteria are inherently resistant to SM treatment [100].

Radiation therapy is a promising candidate for combination treatment with SMs in order to overcome TNF-mediated resistance. In a pre-clinical model of HNSCC, Eytan et al. [101] were able to cure mice using the SM Birinapant and radiotherapy and observed an increase in endogenous TNFα in the tumors. Similar findings supporting a radio-sensitizing role of SMs were observed in different models including NSCLC and Esophageal Squamous Carcinoma (ESCC) using the SM Debio 1143. Encouraging pre-clinical data has been taken forward in HNSCC clinical trials with Birinapant (NCT03803774) and Debio 1143 (NCT02022098). Tao et al. [102] reported increased specific adaptive immunity after treatment with Debio 1143 and ablative radiotherapy (30 Gy) in a LLC-OVA syngeneic model of NSCLC, highlighting a new treatment strategy to increase the immunogenicity of radiation therapy.

#### 5.1.3. Other Immuno-Stimulatory Agents

Similarly, recent preclinical evidence addressing other immuno-stimulatory agents such as TLR (Toll Like Receptors) agonists (TLR2,3,7 and 9) and cytokines (e.g., GM-CSF and FLT-3 ligand) have resulted in some promising preclinical data; however, successful clinical translation has failed to date, partly due to toxicity. TLR agonists are important mediators of inflammatory pathways in the gut, playing a major role in mediating immune responses towards a wide variety of pathogen-derived ligands linking adaptive and innate immunity. Younes et al. [103] showed that immunosuppressive properties of radiation therapy such as recruitment of CD11b+ myeloid cell population can be at least partly overcome by a TLR9 agonist, as those cells express Toll-like-receptors (TLRs). Other immunotherapeutic modalities including cytokines and their inducers, vaccines or adoptive cell transfer (NK cells, DCs, T cells) have been described to augment RT-induced tumor killing.

Intra-tumoral delivery of dendritic cells (DCs) in combination with RT has led to a CD8+ T cell increase in the TILs in a localized prostate cancer model [104] and in tumor-specific immune responses in soft tissue sarcoma [105].

Another explored strategy is the reprogramming of the TME and, more precisely, of Tumor-Associated Macrophages (TAMs). Successful pre-clinical attempts to block M2 polarization by inhibiting STAT3 and STAT6 transcription factors have been reported. Resveratrol was used to block M2 polarization (by decreasing STAT3 activity) and inhibit tumor growth in a mouse xenograft model of lung cancer [106]. However, none of the inhibitors were taken further to clinical studies. Very recently, Lan et al. [107] reported promising data on the simultaneous targeting of TGFβ and PD-L1 with the bispecific antibody Bintrafusp alpha in combination with radiotherapy. The combination resulted in a TME reprogramming and reconstitution of tumor immune-surveillance in poorly immunogenic syngeneic mouse models with very encouraging preclinical results. Additionally, the associated TGFβ sequestration has the potential to result in reduced overlapping toxicities of combination immune-checkpoint inhibition and RT treatment and spare normal tissue toxicity.

### 5.2. Challenges

Despite encouraging combination treatment data, multiple challenges remain.

#### 5.2.1. Dose and Fractionation

One of the challenges is the optimal dose and fractionation of SBRT, as there is currently no international consensus for use in clinical practice. Different regimens have been tested in clinical trials including 30–34 Gy × 1, 15–20 Gy × 3, 12 Gy × 4 and 10–12 Gy × 5 in different settings, making it difficult to favor one single regimen over another. 

Demaria et al. [108] proposed a classification of SBRT regimens into three categories, immunogenic ablative (34 Gy × 1, 18 Gy × 3, 10 Gy × 5), immunomodulatory sub-ablative (8 Gy × 3, 6 Gy × 5) and TME modulatory (0.5 Gy × 4), based on their dominant effects on the crosstalk between the tumor and the immune system. In this way, RT can be viewed as an immunomodulatory tool that can be dispensed or delivered at different doses and fractions according to the nature of the desired immunomodulatory effect to be elicited. 

Designing successful combination treatments with radiotherapy requires understanding precisely how dose and fractionation do matter and accepting that one size dose or regimen do not fit all scenarios. Different fractionation regimens result in distinct immune-modulatory effects, which still remain to be fully deciphered [108]. Considering the dual effects of RT on the host immune system, the RT schedule should be tailored and optimized based on the synergistic effect expected and the immunomodulatory agent used. While aiming at determining one or a range of optimal doses for each type of modulatory agent seeking synergy with RT may seem daunting, it is a necessary task. Similarly, even though synergistic effects with RT have been reported with different agents and tumor models, the mechanisms involved are likely to be different.

Understanding the underlying biology of different doses and fraction regimens of RT is key and can be achieved with adequately designed preclinical research followed by prospective validation in clinical trials.

#### 5.2.2. Sequence of Treatments

Combination treatment can be delivered concurrently to or sequentially to radiation therapy, and once again, the optimal sequence remains to be determined. Considering that different immunomodulatory agents target different pathways and result in different immune changes, careful attention should be paid to the sequencing of treatment in order to elicit the greatest synergistic effect. Preclinical and early clinical data examining both approaches suggest that both sequential and concurrent sequencing are safe and feasible. It is worth nothing that, so far, the optimal sequencing seems to be dependent on the tumor type and the immunotherapy agent being used. Young et al. [109] showed that hypofractionated radiotherapy with anti-CTLA4 worked most effectively when given before irradiation, but anti-OX40 was more effective when administered 1 day after radiation. Another preclinical study by Dovedi et al. [110] showed that PDL-1 inhibition was effective only when given concurrently or at the end of radiation and not one week later.

Several clinical trials that are testing the concurrent approach are ongoing. Pembrolizumab with concurrent SBRT (50 Gy in four fractions) to lung and liver lesions was deemed safe in patients with metastatic NSCLC in a randomized phase I/II trial. [111]. Another single arm I/II trial testing concurrent Ipilimumab commenced with SABR is ongoing [112]. While multiple early-phase data suggest the combination is efficacious and safe, confirmatory large-scale data are eagerly awaited.

Currently the sequential or early sequential approach as seen in the PACIFIC trial is preferred in the treatment of NSCLC [95]. A secondary analysis of the PACIFIC trial points to a better PFS in patients who started Durvalumab within 14 days of chemoradiation completion as opposed to after 14 days. On the other hand, a retrospective analysis of 758 patients who received immunotherapy and radiotherapy within 30 days of each other showed a better overall survival when ICIs were started at least one month before RT [113].

It appears evident that more extensive data on the optimal treatment sequencing are needed, taking into account the types of immunotherapy agents used in selected tumor types. This will enable confirmation of the safety and efficacy of the combination in large cohorts of patients.

#### 5.2.3. How Do We Predict Response?

Predicting the likelihood of individual patients to respond to a radio-immunomodulatory combination is certainly a challenge for the next decade. While some types of biomarkers have been shown to have a predictive value of response to RT, such as components of the DDR machinery [34], genetic [114,115,116], epigenetic signatures [117] and microenvironmental biomarkers [118,119], they have had little impact on the individualized RT treatment delivery, with a few exceptions.

Predictive markers of response to immune-checkpoint blockers have emerged and some are already informing individual decision making in clinical practice, such as PD-L1 expression or the tumor mutational load [120], or DNA mismatch repair (MMR) status [121]. However, markers able to predict the potential synergistic effects of radio-immunotherapy combinations are still in the candidate roles. Expression of the soluble NKG2D (NK-cell-activatory receptor) ligand was described as a potential predictive biomarker candidate. NKG2D was shown to stabilize immunological synapsis between CD8+ CTL and their targets and support adaptive immunity [122]. Radiotherapy promotes exposure of NKG2D ligands on malignant cells, exposing them to NK-cell-dependent lysis or improved recognition by CTLs [123]. In their study, Ruocco et al. [123] showed that the NKG2D ligand is required for synergy between RT and anti-CTLA-4 in 4T1 tumors in vivo.

Another promising approach is the quantification of radiotherapy’s ability to induce a Type 1 interferon response in individual tumors. This could be achieved by measuring levels of TREX1, which has been shown to counteract RT’s ability to drive the secretion of Type 1 IFN by degrading cytosolic dsDNA [124] in ex vivo PDX irradiation models. That would potentially enable dose selection that synergizes with immune checkpoint inhibitors. Recent developments in functional imaging and large scale data analyses using computer algorithms have seen the advent of radiomics [125]. Radiomics allows the extraction of quantitative imaging biomarkers named “radiomic signatures” that most statistically significantly relate to a measured outcome or a tumor biology parameter. A recent review found 43 CT-image based studies, evaluating prognostic or predictive roles of radiomic signatures in NSCLC, that reported at least one positive association between the CT radiomic signature and either outcome or tumor biology [126]. However, the particular radiomic signature derived varies among studies, making a direct comparison difficult. Despite some methodological challenges that need to be addressed before large multicenter studies, imaging biomarkers carry a considerable potential for successful translation.

#### 5.2.4. SBRT-Related Treatment Toxicity

The therapeutic index [127] represents the ratio between the probability of tumor control and that of normal tissue damage or toxicity. SBRT is associated with a steep-dose gradient outside the target volume, thus minimizing the dose to organs at risk and the probability of normal tissue complications. Moreover, advancements in imaging and SBRT delivery including IGRT and IMRT allow a remarkably precise delivery of high dose radiation per fraction to intra-thoracic targets with a good tolerability profile. Nevertheless, toxicities are still reported, ranging from mild fatigue and transient esophagitis to pneumonitis, hemorrhage, chest wall pain, rib fracture or brachial plexopathy [128,129]. Normal tissue toxicity can manifest itself days to years after irradiation, mainly in the heart and lung tissues in patients whose thoracic tumors are irradiated. The most common toxicities include acute pneumonitis, chronic lung fibrosis [130] or radiation-induced heart dysfunction (RIHD) [53,131,132]. Another important manifestation of radiation toxicity is endothelial/vascular injury leading to a loss of endothelial barrier function, resulting in tissue injury [133]. When organs at risk are exposed to sufficiently high doses, the endothelial damage and its associated vascular changes can lead to chronic lesions in those organs [134]. Initial reports suggested an increased risk of toxicity when treating centrally located lung tumors with SBRT, including an 8% risk of death with the 60 Gy in 3 fraction dose [135]. As a result, efforts to sub-classify central lesions and adapt treatment with protracted courses of radiation (5 to 10 fractions) have been made, indicating that SBRT may be safe [136]. However, the results highlight the need for careful attention when selecting patients with ultracentral tumors. An example of the risk-adapted dose fractionation in early-stage NSCLC is 8 × 7 Gy for centrally located tumors [137] and 3 × 18–20 Gy for peripheral lesions. Ultra-central tumors that GTV (Gross Tumor Volume) or PTV (Planned Tumor Volume) directly abuts or overlaps the trachea or proximal bronchial tree still represent a treatment challenge [138], but prospective data from ongoing SUNSET (NCT03306680) and future trials may provide further guidance.

Combination treatment regimens including SBRT and immune-checkpoint inhibitors raise the question of potential additive toxicities and adverse impact on normal tissues [139]. To date, there are few reports describing the toxicity of the combination but prospective data are slowly emerging. Several phase I and II trials investigating the immune-checkpoint inhibitors Pembrolizumab (NCT02608385) Pembrolizumab, Atezolizumab (NCT02400814) and Durvalumab (NCT02904954) in combination with SBRT are on the way. Thus, the importance of accurate preclinical modelling of radiation is key for identifying and preventing normal tissue toxicities. Currently, small animal image-guided conformal irradiators are commercially available and are able to deliver irradiation with a precision close to that used in clinical practice. They also deliver a lower mean dose to surrounding tissues and their use should become standard in studies modelling irradiation in the preclinical setting.

## 6. Conclusions

**Figure 1:**
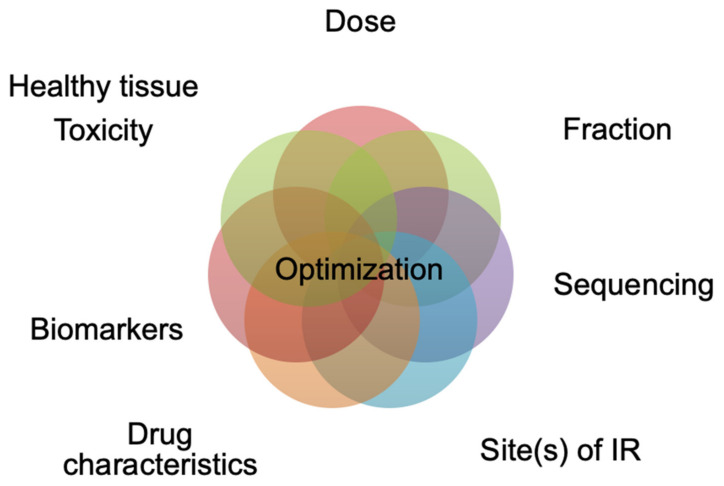
Ongoing challenges of radiotherapy–drug combinations.

The field of radiation oncology has significantly evolved with the advent of SBRT, leading to clinical benefits in the management of NSCLC patients. At the same time, an increasing amount of preclinical and clinical data continues to emerge regarding the combination of radiotherapy and immunomodulatory agents. While the rationale for such combinations is strong and promising, several key factors still need to be fully addressed, such as dose and fractionation, sequencing, selection of the best-suited immunomodulatory agent, toxicities of the combination on healthy tissues and biomarkers to predict responses (Figure 1).

We are starting to acknowledge unique immune-modulatory properties of different dose-fraction regimens and the subsequent need for individually tested combination approaches tailored to the desired synergistic effect. More than ever, one dose or schedule does not fit all clinical scenarios, but neither does it synergize with all immunomodulatory agents. Preclinical models represent an essential tool in the process of harvesting this fundamental understanding and translating it to patient-derived benefits. Clinical benefits derived from recent progress can only be achieved by appropriate and reliable preclinical research mimicking clinical scenarios in almost every aspect: immune status, dose and fraction, radiotherapy delivery modality and planning and tumor microenvironment. Better immunocompetent mouse models of lung cancer are urgently needed to allow faithful study of the tumor–immune interactions and therapeutic modalities relying on those very interactions such as SBRT and immunotherapy. For this reason, orthotropic implantation sites should be systematically used in the currently available immunocompetent syngeneic models. In addition, considering that the majority of preclinical studies looking at synergy between radiotherapy and immunotherapy have been performed in syngeneic ectopic models, GEMMs represent a valuable tool in this setting despite their cited limitations. A further optimization, not achieved yet, would be represented by the development of state-of-the-art humanized PDX models using immunocompetent mice to mimic tumor- and organ-specific microenvironments, especially using orthotropic implantations. 

Due to considerable tumor and micro-environmental heterogeneity, multi-target multi-agent therapeutic strategies are currently being explored and are likely to yield promising results in the near future. One such example is the combination of anti-PD1 and anti-angiogenic agents aiming to alleviate immunosuppression with radiotherapy [140]. Similarly, dual immune-checkpoint blockade including established (anti-PD-1 and anti-CTLA-4) but also new combinations such as Relatlimab (anti LAG-3), as demonstrated in the RELATIVITY-047 (NCT03470922) study on the reinvigoration of T-cell activity, are awaited to be explored in combination with radiation therapy.

While multi-agent multi-modality strategies seem promising, we are a long way from their routine clinical use as a frontline treatment. Not only does the safety profile remain largely unexplored, but as discussed earlier, patient selection, biomarkers, optimal dose and sequencing are all to be addressed. The complexity of any multi-modal combination treatment can be broken down by understanding the immunomodulatory properties of each individual treatment component and how to achieve a balance to make them thrive together. Finally, personalization of radiotherapy is likely to be the cornerstone of the next decade’s progress. Delivering the same dose and fraction to each unique patient and tumor with unique biology and radiation sensitivity is likely to be a distant memory. This cannot be achieved without the development of preclinical models integrating biological and immunological tumor parameters in the dose-fraction equation in order to maximize therapeutic effects. 

## Figures and Tables

**Table 1:** Preclinical models of NSCLC.

Reference	Cell Line	Histology	Implantation Site	Animal Model
Raben et al. [45], McLemore et al. [46]	A549	Adenocarcinoma	s.c. endo bronchial	BALB/cAnNCrlBR athymic (nu+/+) BALB/c or NMRI-nu/nu
Akhtar et al. [47], Chen et al. [48], Wang et al. [49]	H1299	Carcinoma	s.c	Athymic nude mice BALB/c nude
Steiner et al. [50], McLemore et al. [46], Carter et al. [51], Yamori et al. [52]	NCI-H460	Large cell carcinoma	s.c. endo bronchial	athymic nude (Ncr nu/nu)
Steiner et al. [50]	H1975	Adenocarcinoma	s.c.	Athymic nude (nu/nu) NMRI-nu/nu mice
Yamori et al. [52]	NCI-H226	Squamous cell carcinoma	s.c.	BALB/c nude SCID/SCID mice
Akhtar et al. [47], Wang et al. [53]	HCC827	Adenocarcinoma	s.c. orthotopic	Athymic nu/nu BALB/cA nude mice
Lam et al. [54]	HCC4006	Adenocarcinoma	s.c.	Nude BALB/cAnN-nu
Zimonjic et al. [55], Steiner et al. [50], Lam et al. [54], Onn et al. [56]	NCI-H358	Broncho alveolar carcinoma	s.c. orthotopic	Athymic nu/nu mice BALB/c nude Athymic nude
Doki et al. [57], Mordant et al. [58]	LLC	Lewis Lung carcinoma	Orthotopic	C57BL/6 mice
Yamori et al. [52]	NCI-H23	Adenocarcinoma	s.c.	BALB/c nude mice
Williams et al. [25]	TL-1	Squamous cell carcinoma	s.c.	CB-17 scid/scid mice
Takahoshi et al. [59]	NCI-H441 and H440	Adenocarcinoma	Orthotopic	Athymic nude

s.c.: subcutaneous.

**Table 2:** Summary of advantages and limitations of the main preclinical models used in cancer research.

Models	Advantages	Limitations
Cell line models (2D)	Easy and widely available wide range of tumor models	Fail to reproduce tumor heterogeneity Do not reflect original tumor biology
Organoids	Simple Mass production Co-culture possible	Difficult long-term culture Low throughput
Patient-derived tumor xenograft models (PDX)	Reproduce heterogeneity of human disease	Immune-deficient hosts Vasculature and stroma of murine origin Low implantation rate
Humanized patient-derived xenografts	Robust human immune system engraftment Resemblance of tumors to human donor	Requires autologous immune reconstitution Residual mouse innate immunity Cost and infrastructure
Syngeneic mouse models	Immunocompetent host Evaluation of targeted therapies and toxicity Good concordance in drug response Ease of manipulation Rapid growth and reproducible	Lack of native tumor microenvironment Lack of heterogeneity Few host strains Limited number of transplantable cell lines
Genetically Engineered Mouse Models (GEMMs)	Study of the role of specific mutations in cancer development and progression Native microenvironment Variety of genetic backgrounds possible	Slow tumor development Simultaneous study of a limited number of genes Tumor and TME of murine origin Frequent multiple simultaneous tumors Breeding challenges
